# Oral care knowledge, attitude and practice among nursing staff in acute hospital settings in Hong Kong

**DOI:** 10.1371/journal.pone.0289953

**Published:** 2023-08-15

**Authors:** Pui Ki Tsui, Pui Hing Chau, Janet Yuen Ha Wong, Man Ping Wang, Xiaoli Gao, Otto Lok Tao Lam, Katherine Chiu Man Leung, Edward Chin Man Lo, Agnes Tiwari

**Affiliations:** 1 School of Nursing, The University of Hong Kong, Hong Kong Special Administrative Region, Hong Kong, China; 2 Department of Childcare, Elderly and Community Services, Hong Kong Institute of Vocational Education (Sha Tin), Hong Kong Special Administrative Region, Hong Kong, China; 3 School of Nursing & Health Studies, Hong Kong Metropolitan University, Hong Kong Special Administrative Region, Hong Kong, China; 4 Faculty of Dentistry, National University of Singapore, Singapore, Singapore; 5 Saw Swee Hock School of Public Health, National University of Singapore, Singapore, Singapore; 6 George & Fay Yee Centre for Healthcare Innovation, Max Rady College of Medicine, Rady Faculty of Health Sciences, University of Manitoba, Winnipeg, Manitoba, Canada; 7 Faculty of Dentistry, The University of Hong Kong, Hong Kong Special Administrative Region, Hong Kong, China; 8 School of Nursing, Hong Kong Sanatorium & Hospital Limited, Hong Kong Special Administrative Region, Hong Kong, China; Hosei University: Hosei Daigaku, JAPAN

## Abstract

Investigating the oral care delivered by nursing staff in acute hospital setting is having a remarkable shortage within the current literature. This was provoked due to lack of previous performed investigation in the acute hospital setting besides inconsistent existence of a standardized and comprehensive oral care knowledge, attitude and practice (KAP) instrumentation. Therefore, the purpose of this study is to assess the oral care KAP level for inpatients among nursing staff; to identify possible barriers to the provision of oral care; and to identify training preferences to improve the oral care of inpatients, in acute hospital settings in Hong Kong; and to provide standardized comprehensive KAP based assessment tool that would benefit and guide other future studies. In this study, a cross-sectional survey was conducted after a 55-item self-administered structured questionnaire was developed. A modified KAP tool was developed. The tool includes 4 domains: oral care knowledge, attitude, practice, and experience. Nursing staff was recruited from July 2018 to April 2019 via convenience sampling. Either online or printed questionnaires were completed. Proportions of nursing staff with good KAP, as defined by having 60% of the total score in the respective domain, were estimated with 95% confidence intervals (CI). Analysis of covariance was used to compare the mean scores of KAP among different independent variables and identify the factors associated with good KAP. 404 nursing staff were recruited. Approximately 29.5%, 33.7% and 14.9% of the respondents had good oral care knowledge, attitude and practice, respectively, and 53.2% of the respondents had unpleasant oral care experience. Better oral care practice was associated with higher levels of oral care knowledge (β = 0.1) and oral care attitude (β = 0.3). To conclude: nursing staff in acute hospital settings reported low levels of oral care KAP with variations between the RN, EN and HCA. This study adds to the literature the association between oral care unpleasant experiences and the oral care practice, as well as oral care knowledge and attitude which also in turns associated with practice. The developed standardised tool could be applied for future studies. Recommendations on the future research, training and practices were made.

## Introduction

Oral care is provided and maintained by nursing staff to those who are ill or unable to perform oral care by themselves, and it can be regarded as a fundamental aspect of care. It involves different procedures, for example, tooth brushing, oral health assessment and examination for individuals with different conditions to maintain oral health [1, 2]. Medical nurses can identify oral health problems through assessment, and arrange referrals to other health care specialists such as dentists for intervention as necessary [3].

The World Health Organization (WHO) [4] advocates and encourages relevant health policies to improve and promote oral care to patients. Improved oral care and health can suppress respiratory pathogen colonisation in the oropharynx, reducing infection and other respiratory diseases and improving morbidity and mortality rates [5]. It also reduces the incidence of ventilator-associated pneumonia in intensive care units (ICUs) [6]. Therefore, poor oral health would lead to oral diseases [7] which in turn contributing to higher health and economic burden [8].

Oral diseases are the most prevalent non-communicable diseases in the world, with a prevalence rate of 45% [9]. It can contribute to pain, discomfort and even death of individuals. The Global Burden of Disease 2015 Study stated that approximately 3.5 billion people globally are suffering from poor oral health, as compared to 1.0 billion people who are affected by mental disorder and 0.5 billion by diabetes mellitus [9, 10]. However, despite its importance, oral health is usually being neglected [11–13].

Provision of good oral care in the hospital setting largely relies on the knowledge, attitude and practice (KAP) of nursing staff serving at different rank and positions. To date, most studies about oral care KAP of nursing staff were conducted in ICUs and not in acute hospital settings, such as acute medical and surgical or general hospital settings [14–17]. In Hong Kong, only one qualitative study interviewed ten ICU nurses and reported a relatively low priority of oral care procedures compared with other nursing care procedures [16]. Moreover, there was also a lack of standardized tools to assess KAP which made comparisons across studies difficult.

## Materials and methods

This study used a cross-sectional design employing a self-administered structured questionnaire. The aims were to assess the level of KAP of oral care for inpatients among nursing staff; to identify possible barriers to the provision of oral care; to identify training preferences to improve the oral care of inpatients, in acute hospital settings in Hong Kong, and to provide standardized comprehensive KAP based assessment tool that would benefit and guide other future studies.

### Study population

Nursing staff who provided routine oral care in acute hospital settings in Hong Kong from July 2018 to April 2019 were recruited by convenience sampling. In Hong Kong, there are three types of nursing staff, namely registered nurses (RN), enrolled nurses (EN) and health care assistants (HCA). The roles of RN are to provide holistic and comprehensive care to patients, whereas EN have to delegate more fundamental nursing care [18, 19]. As for the roles of HCA, including nursing assistants, they only receive basic training and are mainly to assist both RN and EN provide nursing care to patients.

The inclusion criteria were: 1) nursing staff (RN, EN and HCA); 2) working at public or private hospitals in acute settings; and 3) with patients in the participants’ ward who needed nursing staff to perform oral care. The patients’ need for oral care was assessed, determined and initiated by nursing staff and not upon the request of patients. Nursing staff working at homes for the aged, non-acute hospitals and non-acute wards, such as palliative, rehabilitation and outpatient departments, were excluded.

With the assumption of 50% true proportion of good practice, a margin of error of 5%, a 95% confidence level, and 5% incomplete questionnaires, a sample of 402 nursing staff was required. There were approximately 42,485 RN and 14,238 EN in Hong Kong in 2018 [20]. Since a concrete figure on HCA was unavailable, we adopted the same ratio of 3:1 for RN to EN and RN to HCA in the sample.

### Data collection

The data were collected through convenience, snowball and quota sampling. Moreover, mixed-mode survey (both online and printed version), which can enhance time efficiency, lower cost, increase response rate, and maximize the inclusion of heterogeneous samples, was adopted [21]. For online version, online Survey Software (Qualtrics) (http://www.qualtrics.com/) was used. The survey link was delivered through bulk email system of the university and social media. For the printed survey, a sealed package enclosed with information sheet, printed copy of questionnaire and return envelope was prepared. No material incentives were provided. The duration of the survey was approximately 15 min. Test-retest reliability was performed with 30 participants who completed the questionnaire again after one week.

### Ethical consideration

Informed consent was obtained from the participants and the study was conducted with consenting participants. This study was conducted after obtaining institutional review board approval (UW 16–197).

### Instrument

**Fig 1:**
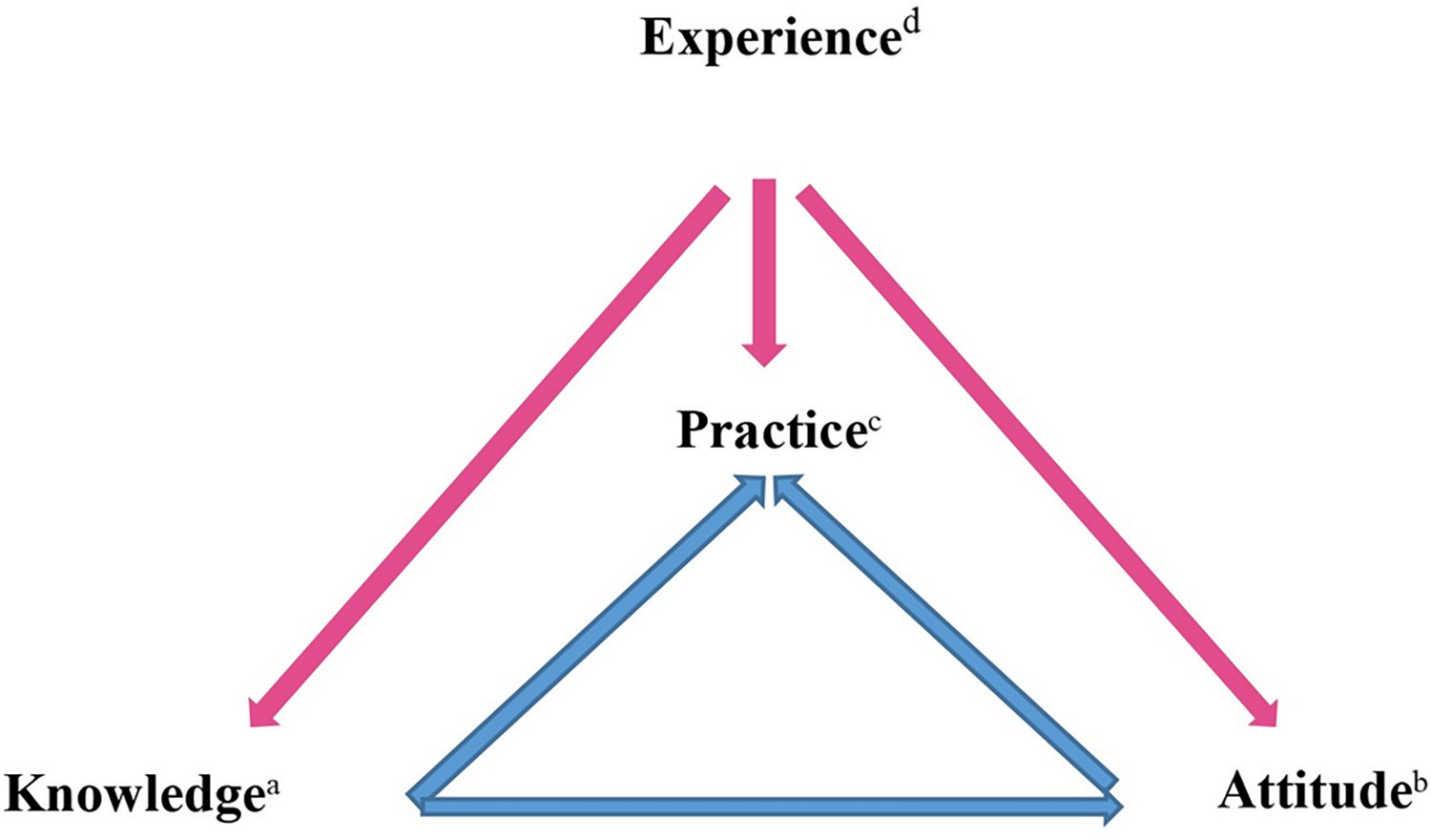
Conceptual model of Knowledge, Attitude and Practice (KAP). (a) Knowledge: knowledge about the kinds of patients with oral care cleansing needs, indicators of poor oral health, adverse effects of poor oral care, adverse drug effects on oral health, familiarity or use of oral care assessment scale and benefits of oral care practice. (b) Attitude: oral care priority of nursing staff and reasons for performing oral care. (c) Practice: actual practice evaluated in terms of the frequency of oral care, duration of oral care cleansing, use of cleansing agents and solutions, other types of oral care, provision of oral health assessment and use of oral care guidelines or protocols. (d) Experience: unpleasant experience, training experience and years of work experience.

The KAP model was adopted as the conceptual framework to guide the instrument development [4, 22]. The KAP model has been used for oral care assessment and education to identify nursing professionals’ knowledge and attitude about oral care to help elucidate the reasons for their attitude and practice [23–29]. Meanwhile, nurses with more critical care experience performed oral care more often than those with less experience [13], and nurses experienced unpleasant resistant behaviour from patients regarding oral care [30]. Therefore, experience was assumed to influence the oral care knowledge and attitude and hence the oral care practice of the nursing staff, and a new element (experience) was proposed to the conceptual framework (Fig 1).

Based on similar studies [23, 25–28] and local focus groups conducted by the team, a structured questionnaire was developed. Six experts including three academic staff (two nurses and one dentist) and three frontline health care providers (nurse, dentist and doctor) reviewed the questionnaire and assessed its content validity. Item content validity index (I-CVI) was calculated by the number of experts rating the item as very relevant or relevant divided by the total number of experts. A pilot study was carried out with 15 nursing staff (9 RN, 3 EN and 3 HCA). S1 Appendix presents the development process of the questionnaire.

The final questionnaire had one screening section and six parts (S2 Appendix). The six parts were i) practice of oral care for frail patients in hospitals, ii) knowledge of oral care, iii) attitude towards the oral care of inpatients, iv) training about oral care, v) oral care experience and vi) the profile of the nursing staff. All items had I-CVI at least 0.83, indicating good content validity. The intra-class correlation coefficient for oral care practice score, knowledge score and attitude score was 0.95, 0.97 and 0.95 respectively, showing good test-retest reliability.

#### i) Oral care practice

Oral care practice was the primary outcome. Good practice in each aspect was defined according to the guidelines from both local and international guidelines [1, 31–33]. Nine out of the 14 items were used to calculate the composite score (Table 1). These items were selected because they were widely adopted in the literature for oral care practice [12, 13, 23, 26, 27, 34–39]. A score of 1 was given to good practice and 0 otherwise, giving a composite score ranging from 0 to 9, with higher score indicating better practice. Based on the score classifications of oral care knowledge from previous studies [34, 40], ≥60% of the total score was regarded as moderate-to-good oral care practice. Therefore, a score of ≥6 out of 9 were regarded as good practice. The remaining questions were included to assess oral care practice from different perspectives.

**Table 1:** Calculation of composite score for oral care practice.

Item no	Questions	Scoring for the composite score
1	Frequency of oral cleansing	<Twice daily: 0 ≥Twice daily: 1
3	Time needed on each oral cavity cleansing	<2 minutes or >5 minutes: 0 2 to 5 minutes: 1
4	Tools used for oral care	“Toothbrush” unselected: 0 “Toothbrush” selected: 1
5	Agents used for oral care	“Toothpaste” unselected: 0 “Toothpaste” selected: 1
6	Other types of oral care provided besides oral cavity cleansing	“Denture cleansing” unselected: 0 “Denture cleansing” selected: 1
7	Provision of oral health assessment	“No”/ “Yes, always” unselected: 0 “Yes, always” selected: 1
8	When to provide oral health assessment	“Admission” unselected: 0 “Admission” selected: 1
11	Provision of guidelines or protocols for oral care procedures	“Yes” unselected: 0 “Yes” selected: 1
12	Use of oral care guidelines or protocols	“Yes” unselected: 0 “Yes” selected: 1

#### ii) Oral care knowledge

Oral care knowledge was the secondary outcome, 6 out of the 8 items were used to calculate the composite score (Table 2). These items were selected since they were widely covered in the literature on oral care knowledge [12, 23, 34, 35, 41, 42]. Following the previous study [26], one mark would be deducted for each selected incorrect option about adverse effects of poor oral health. Therefore, the composite score ranged from -2 to 30, with higher scores indicating better oral care knowledge. Based on the score classifications of oral care knowledge from previous studies [34, 40], ≥60% of the total mark was regarded as moderate-to-good oral care knowledge. Therefore, a score of ≥18 out of 30 were regarded as good knowledge. The remaining items were included to assess confidence level of the nursing staff.

**Table 2:** Calculation of composite score for oral care knowledge.

Item no	Questions	Scoring for the composite score
1	Kinds of patients requiring oral care	1 for each correct option Range: 0 to 5
2	Indicators of poor oral health	1 for each correct option Range: 0 to 5
3	Possible adverse effects of poor oral health	1 for each correct option -1 for each wrong option Range: -2 to 5
4	Adverse drug effects on oral health	“No”: 0 “Not sure”: 3 “Yes”: 5
5	Use of or familiarity with oral care assessment scales	"No”: 0 “Yes, heard”: 3 “Yes, used”: 5
8	Possible benefits of oral care practices	1 for each correct option Range: 0 to 5

#### iii) Oral care attitude

Oral care attitude was another secondary outcome, which was a single item on oral care priority [25,26]. The responses were in the form of a 10-point scale ranging from 0 to 10, a high score indicated a more positive attitude. In accordance with the method of previous literature [40] and to achieve consistency in the classification of good practice and knowledge, ≥60% of the total mark was adopted for the classification of good attitude. Therefore, a score of ≥6 out of 10 was regarded as good attitude. Remaining items were included to assess oral care attitude from different perspectives.

#### iv) Training about oral care

Six questions were used to investigate training about oral care. However, composite score was not calculated.

#### v) Oral care experience

Twelve questions were used to investigate oral care experience. The single question on the hindrance was based on a five-point Likert scale and those opted “totally hindered” and “somewhat hindered” were considered as having unpleasant experience hindrance.

#### vi) Profile of the nursing staff

Twelve questions were used to collect demographic information, such as present work position (i.e., RN, EN or HCA), first and latest qualification, years of qualification, highest education level, current work setting, shift arrangement, years of work experience and funding mode of the current work setting.

### Statistical analysis

The data were analysed using the SPSS version 25.0 (IBM Corp, 2019). Descriptive statistics were used to summarise the socio-demographic characteristics and the outcome measures. The proportion of participants with good practice, good knowledge and good attitude were presented with a 95% confidence interval (CI). Chi-square test was conducted to identify difference in such proportions among RN, EN and HCA. One-way ANOVA was performed to identify difference in the scores of oral care practice, knowledge and attitude among the three groups of nursing staff.

Analysis of covariance (ANCOVA) was used to investigate the factors of oral care KAP scores. In the model of knowledge, the independent variables were a set of demographic variables, including work position, education level, work settings, years of work experience, oral care training and unpleasant experience. In the model of attitude, besides the demographic variables, the independent variables included the knowledge score. For the model of practice, besides the demographic variables, the independent variables included the knowledge and attitude scores. Backward elimination was adopted to select the final models. The cases with missing information were excluded in the respective analyses. Multicollinearity was detected by Variance Inflation Factor (VIF)>5 [43]. The level of significance was set at 0.05.

## Results

### Participants’ characteristics

**Fig 2:**
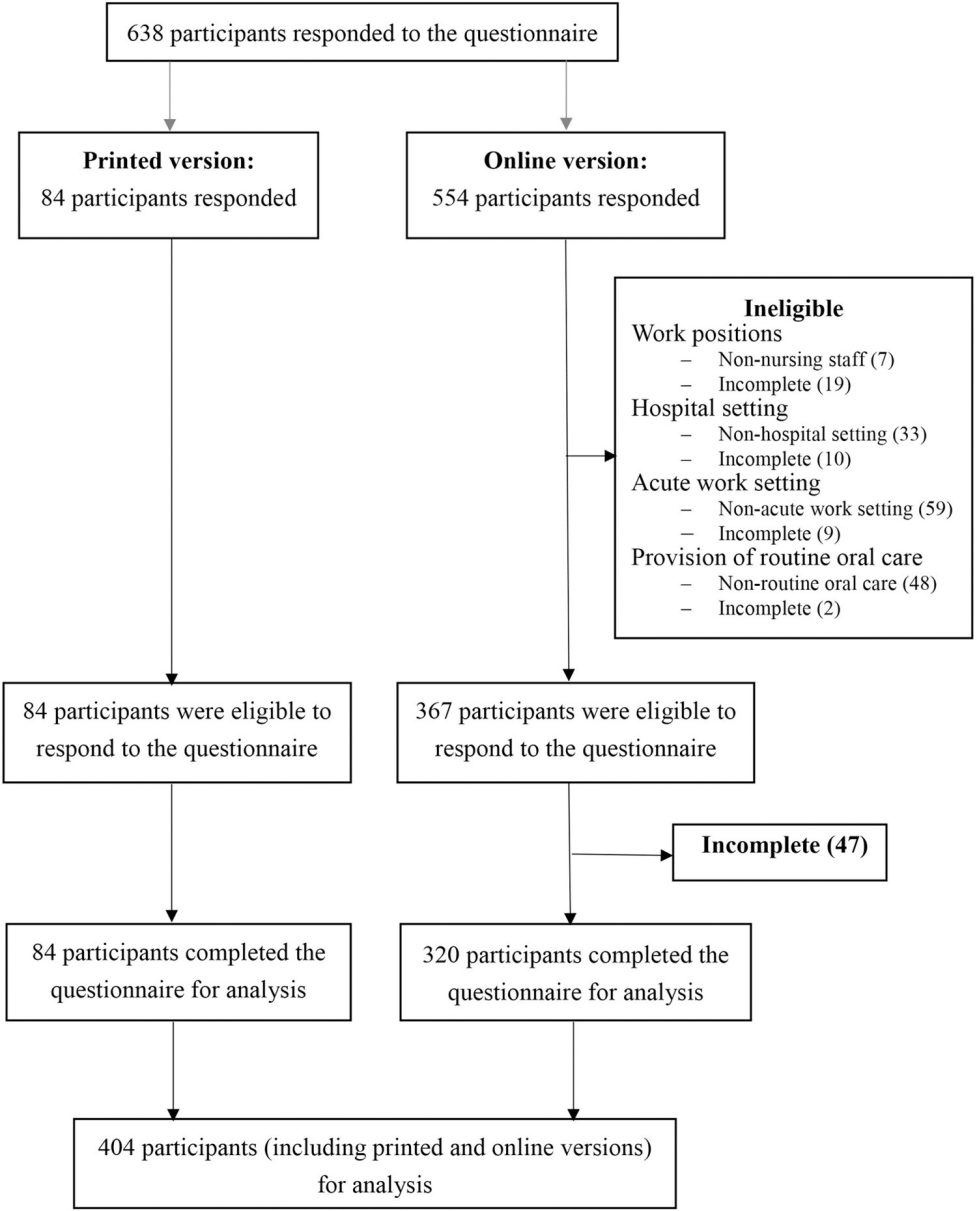
Flow of participants for their eligibilities to the questionnaire.

A total of 638 participants (84 from paper survey and 554 from online survey) responded to the questionnaires from July 2018 to April 2019. After excluding ineligible cases (Fig 2), 404 respondents (54.5% were RN, 20.8% were EN and 24.8% were HCA) were included in the analysis. Table 3 shows the respondents’ characteristics, with 29.4% having a sub-degree, 40.8% worked in acute medical units, 98.3% had to work in shifts with internal rotation, 59.4% had 0–5 years of work experience, and 76.4% worked in public hospitals.

**Table 3:** Respondents’ characteristics.

Study Sample (*n* = 404)	%	*N*
Work position		
Registered nurses	54.5	220
Enrolled nurses	20.8	84
Health care assistants	24.8	100
Education		
Master’s degree and above	24.1	97
Bachelor’s degree	29.1	117
Sub-degree	29.4	118
Diploma/certificate/other	17.4	70
Work settings		
Medical e.g., stroke, cardiology	40.8	164
Surgical e.g., orthopaedic	36.8	148
Intensive care unit	12.2	49
Other e.g., oncology and psychiatry	10.2	41
Shift arrangement		
Internal rotation	98.3	395
Other e.g., long shift and day duty	1.7	7
Years of work experience		
0–5 years	59.4	238
6–10 years	29.2	117
11 years or above	11.4	46
Types of hospitals		
Public	76.4	307
Private	23.6	95

### Oral care practice

Overall mean score of oral care practice was 3.4 (95% CI: 3.2 to 3.6) with the highest mean score of 4.1 in EN (95% CI: 3.7 to 4.5), followed by 3.4 in RN (95% CI: 3.1 to 3.7) and 2.7 in HCA (95% CI: 2.4 to 3.0) (between-group difference: p<0.001). Only 14.9% (95% CI: 11.4% to 18.4%) of them were rated as having good performance in oral care practice (i.e., a practice score of ≥6 out of 9). When analysed by work position, 16.4% (95% CI: 11.5% to 21.3%) of RN, 20.2% (95% CI: 11.6% to 28.8%) of EN and 7.0% (95% CI: 2.0% to 12.0%) of HCA had good oral care practice with significant between-group difference (p = 0.027) (Table 4).

**Table 4:** Summary results of oral care practice.

	Oral Care Practice Score		Proportion of Good Practice	
	Mean	95% CI	Proportion (%)	95% CI
RN	3.4	3.1 to 3.7	16.4	11.5% to 21.3%
EN	4.1	3.7 to 4.5	20.2	11.6% to 28.8%
HCA	2.7	2.4 to 3.0	07.0	2.0% to 12.0%
Overall	3.4	3.2 to 3.6	14.9	11.4% to 18.4%
Between group difference	p<0.001	-	p = 0.027	-

**Fig 3:**
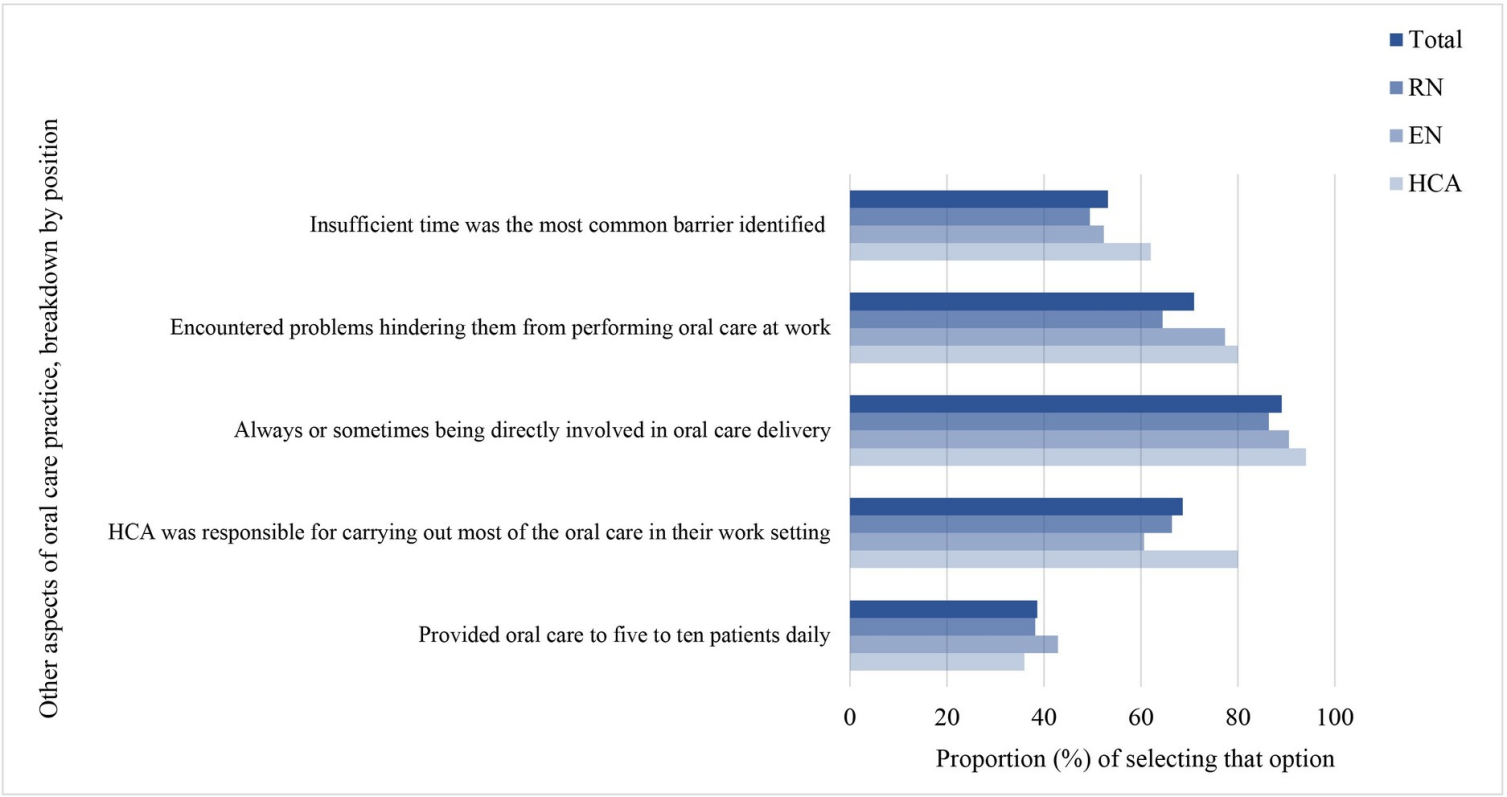
Other aspects of oral care practice, breakdown by position. Bars showing the proportion of respondents engaging in the specified practice. Dark blue bar for overall, the lighter blue bars for the breakdowns by RN, EN and HCA.

Fig 3 shows the other aspects about oral care practice for the participants overall and breakdown by position. Besides, usefulness of oral care guidelines for improving oral care practice was perceived to be neutral. The mean usefulness score was 5.4 out of 10, with the highest mean score observed in RN (5.6), followed by EN (5.5) and HCA (5.0).

### Oral care knowledge

The overall mean score of oral care knowledge was 14.1 (95% CI: 13.6 to 14.6), with the highest mean score of 16.6 in RN (95% CI: 16.0 to 17.2), followed by 13.1 in EN (95% CI: 12.1 to 14.1) and 9.6 in HCA (95% CI: 8.6 to 10.6) (between-group difference: p<0.001). Approximately 29.5% (95% CI: 25.1% to 33.9%) of the respondents were regarded as having good knowledge in oral care practice (i.e., a knowledge score of ≥18 out of 30). Among the RN, EN and HCA, 44.1% (95% CI: 37.5% to 50.7%), 19.0% (95% CI: 10.6% to 27.4%) and 6.0% (95% CI: 1.3% to 10.7%) had good oral care knowledge, respectively, with significant between-group difference (p<0.001) (Table 5).

**Table 5:** Summary results of oral care knowledge.

	Oral Care Knowledge Score		Proportion of Good Knowledge	
	Mean	95% CI	Proportion (%)	95% CI
RN	16.6	16.0 to 17.2	44.1	37.5% to 50.7%
EN	13.1	12.1 to 14.1	19.0	10.6% to 27.4%
HCA	9.6	08.6 to 10.6	06.0	1.3% to 10.7%
Overall	14.1	13.6 to 14.6	29.5	25.1% to 33.9%
Between group difference	p<0.001	-	p<0.001	-

Overall mean confidence score in performing oral care procedures for patients was 6.2 with the highest mean score of 6.6 obtained among RN, followed by EN (6.1) and HCA (5.4). As for the mean of confidence in answering questions about oral care from patients or their families, the overall mean score was 5.7 with the highest mean score of 6.3 obtained among RN, followed by EN (5.5) and HCA (4.4).

### Oral care attitude

**Fig 4:**
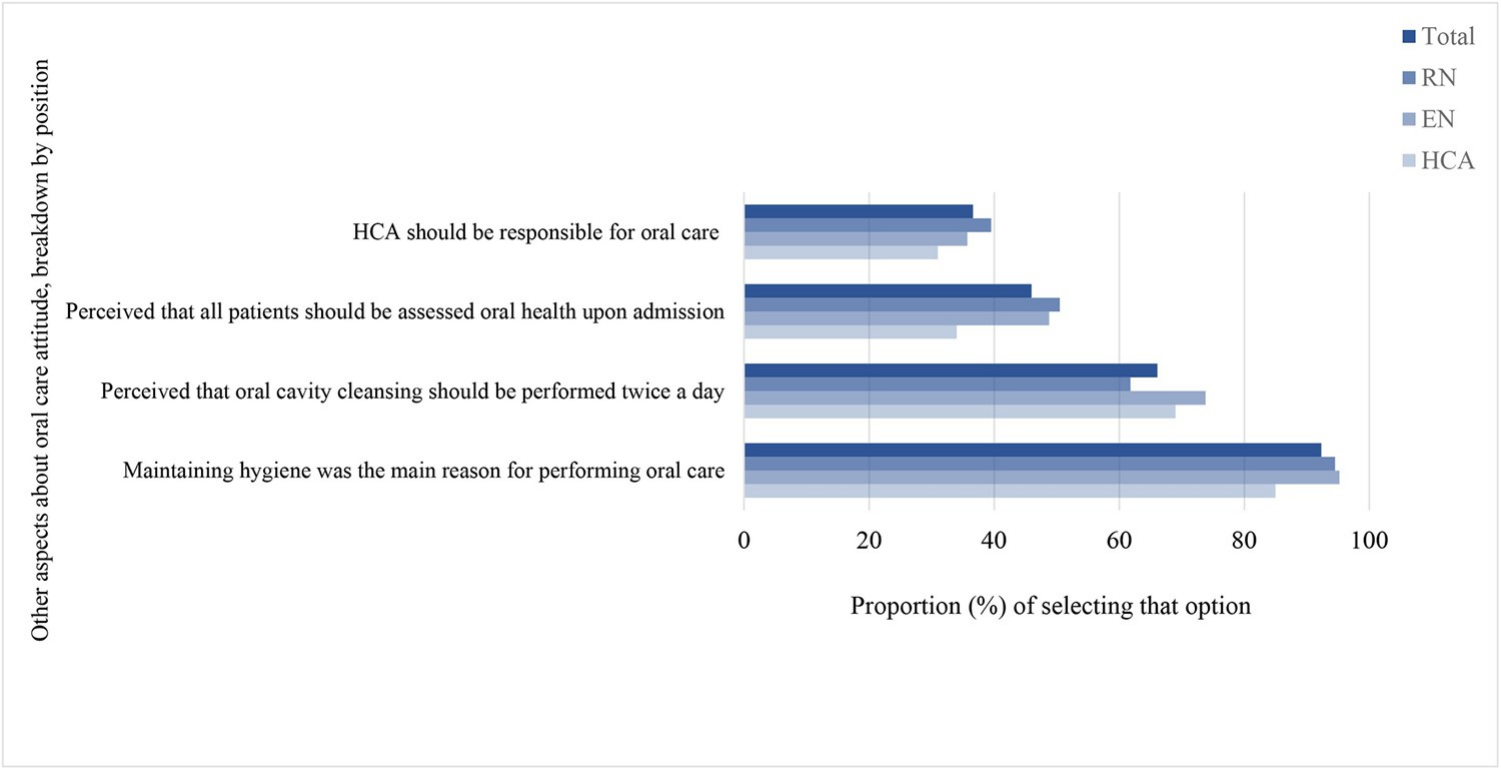
Other aspects about oral care attitude, breakdown by position. Bars showing the proportion of respondents about other aspects of oral care attitude. Dark blue bar for overall, the lighter blue bars for the breakdowns by RN, EN and HCA.

The overall means score of oral care attitude was 4.5 (95% CI: 4.3 to 4.7), with the highest mean score of 5.0 in EN (95% CI: 4.7 to 5.4), followed by 4.3 in HCA (95% CI: 4.0 to 4.7) and 4.3 in RN (95% CI: 4.0 to 4.6) (p = 0.01 for between-group difference). Approximately 33.7% (95% CI: 29.1% to 38.3%) of the participants were rated as having good attitude towards oral care practice (i.e., an attitude score of ≥6 out of 9). Among the EN, RN and HCA, 51.2% (95% CI: 40.5% to 61.9%), 31.8% (95% CI: 25.6% to 38.0%) and 23.0% (95% CI: 14.8% to 31.2%) had good oral care attitude, respectively with significant between-group difference (p<0.001). (Table 6) Fig 4 shows the other aspects about oral care attitude for the participants overall and breakdown by position.

**Table 6:** Summary results of oral care attitude.

	Oral Care Attitude Score		Proportion of Good Attitude	
	Mean	95% CI	Proportion (%)	95% CI
RN	4.3	4.0 to 4.6	31.8	25.6% to 38.0%
EN	5.0	4.7 to 5.4	51.2	40.5% to 61.9%
HCA	4.3	4.0 to 4.7	23.0	14.8% to 31.2%
Overall	4.5	4.3 to 4.7	33.7	29.1% to 38.3%
Between group difference	p<0.001	-	p<0.001	-

### Oral care training

**Fig 5:**
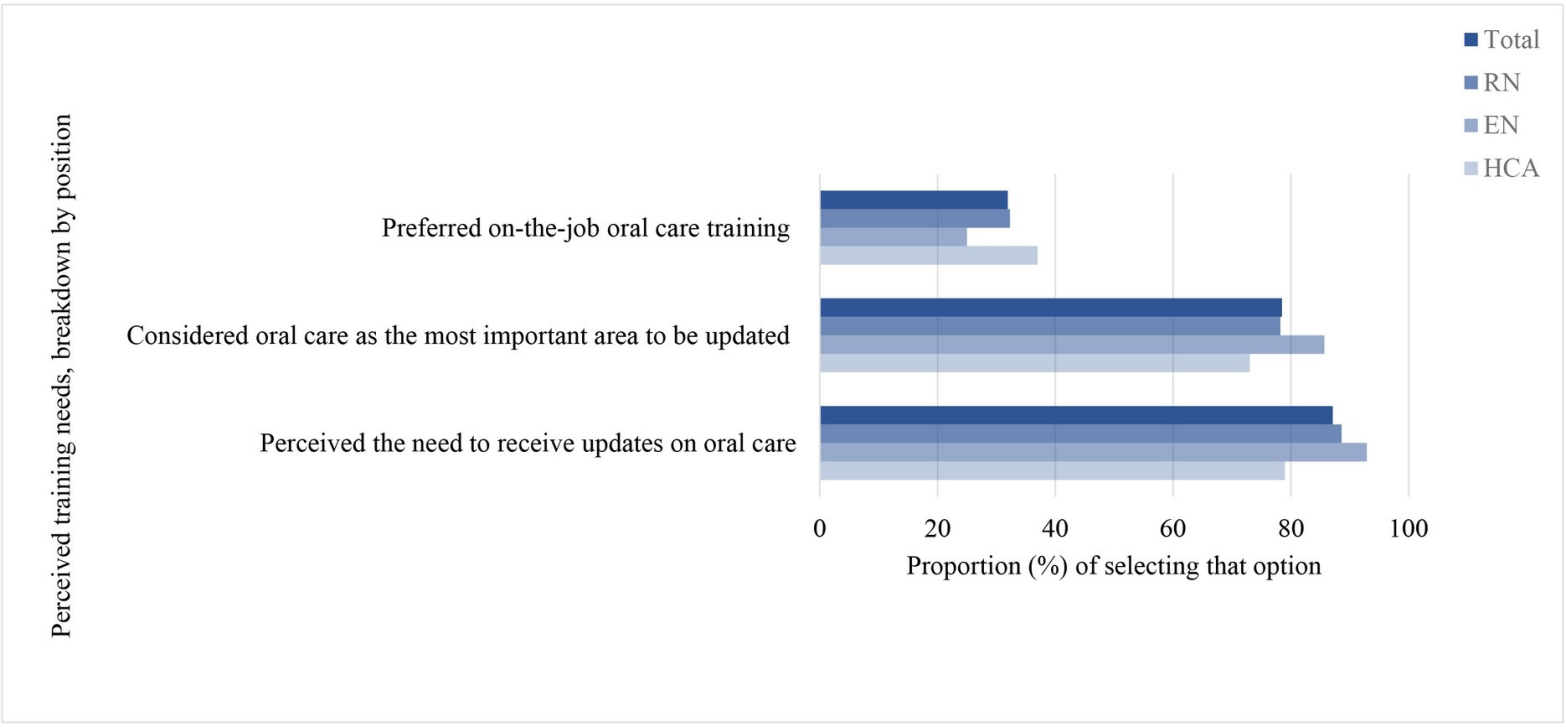
Perceived training needs, breakdown by position. Bars showing the proportion of respondents with perceived training needs. Dark blue bar for overall, the lighter blue bars for the breakdowns by RN, EN and HCA.

Approximately 61.8% (RN: 80.0%, EN: 84.5% and HCA: 3.0%) of the nursing staff received oral care training at nursing school. Approximately 87.1% of them perceived the need to receive updates on oral care at yearly intervals (52.3%). Oral care procedure (78.5%) was considered as the most important area to be updated. They preferred on-the-job training (31.9%). Fig 5 shows the training needs for the participants overall and breakdown by position.

### Oral care experience

**Fig 6:**
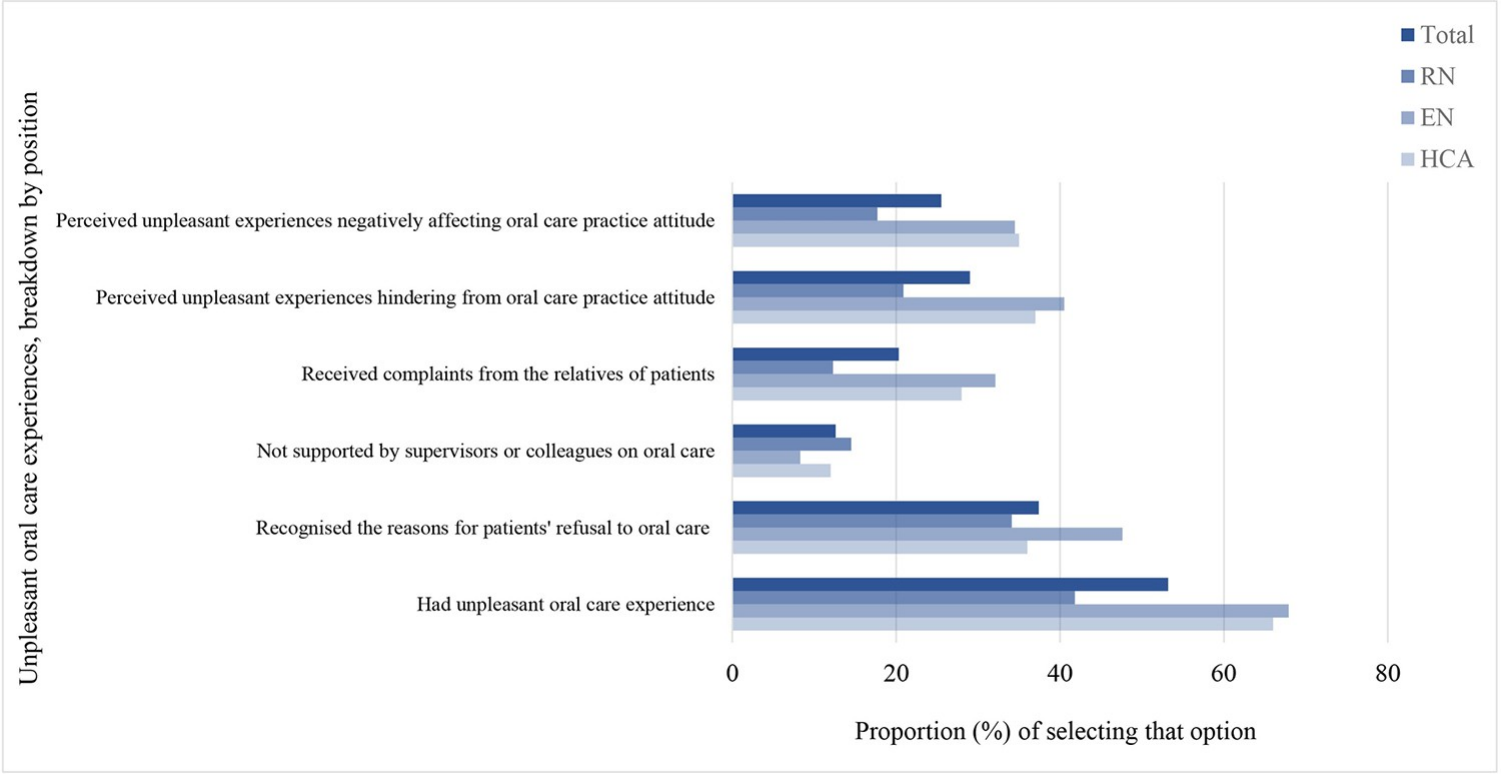
Unpleasant oral care experiences, breakdown by position. Bars showing the proportion of respondents with particular oral care experiences. Dark blue bar for overall, the lighter blue bars for the breakdowns by RN, EN and HCA.

More than half of the participants (53.2%, RN: 41.8%, EN:67.9% and HCA:66%) had unpleasant oral care experience, and non-compliance from patients was the most common one (36.9%). Fig 6 shows the details of the unpleasant oral care experiences for the participants overall and breakdown by position.

### Factors for oral care knowledge, attitude and practice

Table 7 presents the final models of ANCOVA with oral care KAP scores. The model for oral care knowledge had a R^2^ of 0.3, a significant relationship was found among knowledge and work positions (p<0.001), oral care guideline training (p = 0.001), unpleasant work experience (p = 0.028) and work settings (p<0.001). For work positions, HCA (β = -6.9, 95% CI: -8.0 to -5.8, p<0.001) and EN (β = -3.7, 95% CI: -4.9 to -2.5, p<0.001) had less oral care knowledge compared with RN. For oral care guideline training, those who did not receive training on oral care guidelines showed less oral care knowledge compared with those who did (β = -1.5, 95% CI: -2.4 to -0.6, p = 0.001). Compared to those with unpleasant oral care experience, those without had less oral care knowledge (β = -1.1, 95% CI: -2.0 to -0.1, p = 0.028). Regarding work settings, better oral care knowledge was reported in those assigned to units other than medical, stroke and geriatric departments (β = 2.3, 95% CI: 0.7 to 3.9, p = 0.004).

**Table 7:** Final model of analysis of covariance for oral care knowledge, attitude and practice with independent variables.

	Knowledge (n = 402)^c^					Attitude (n = 402)^c^					Practice (n = 404)^c^					
Independent variables	β	95% CI			p-value	β	95% CI			p-value	β	95% CI			p-value	
Work positions					<0.001										<0.001
RN (ref)	0					-	-		-	-	0				
EN	-3.7	-4.9	to	-2.5	<0.001	-	-		-	-	-0.9	0.4	to	-1.3	<0.001
HCA	-6.9	-8.0	to	-5.8	<0.001	-	-		-	-	-0.1	-0.4	to	-0.5	<0.805
Education										<0.001					
Master or above (ref)	-	-		-	-	0					-	-		-	-
Degree	-	-		-	-	-0.8	-1.3	to	-0.3	<0.001	-	-		-	-
Sub-degree	-	-		-	-	-0.4	-0.1	to	-0.9	<0.117	-	-		-	-
Diploma/cert	-	-		-	-	-0.1	-0.7	to	-0.5	<0.804	-	-		-	-
Work settings					<0.001					<0.001					
Medical (ref)	0					0					-	-		-	-
Surgical	-0.9	-1.9	to	-0.1	<0.082	-0.2	-0.2	to	-0.6	<0.224	-	-		-	-
ICU	-1.3	-0.2	to	-2.7	>0.094	-1.7	1.1	to	-2.2	<0.001	-	-		-	-
Other	-2.3	0.7	to	-3.9	>0.004	-0.7	0.1	to	-1.3	<0.022	-	-		-	-
Oral care guideline training															
Yes (ref)	0					0					0				
No	-1.5	-2.4	to	-0.6	>0.001	-0.6	-0.9	to	-0.2	<0.001	-0.6	-1.0	to	-0.3	<0.001
Unpleasant experience															
Yes (ref)	0					0					-	-		-	-
No	-1.1	-2.0	to	-0.1	<0.028	-0.5	-0.8	to	-0.1	<0.011	-	-		-	-
Knowledge^a^	NA					-0.1	0.0	to	-0.1	0.005	-0.1	0.1	to	-0.1	<0.001
Attitude^b^	NA					NA					-0.3	0.2	to	-0.4	<0.001

^a^Knowledge scores ranged from -2 to 30, and higher scores indicated higher level of oral care knowledge.

^b^Attitude scores ranged from 0 to 10, and higher scores indicated higher level of oral care attitude.

^c^Cases with missing independent variables were excluded from the respective model.

- Not selected by the backward elimination.

5<VIF<7.3 for work positions and education. VIF<2 for other variables.

NA Not included in the full model.

In the model of oral care attitude (R^2^ = 0.2), a significant relationship was shown with education (p<0.001), work settings (p<0.001), oral care guideline training (p = 0.001), unpleasant experience (p = 0.011) and oral care knowledge (p = 0.005). Work position was eliminated from the model by the backward elimination, yet there might be concerns about multicollinearity as the VIF of work position and education was greater than 5. Compared to those with unpleasant oral care experience, those without had poorer oral care attitude (β = -0.5, 95% CI: -0.8 to -0.1, p = 0.011). Regarding education, compared to those at master’s degree level, bachelor’s degree holders (β = -0.8, 95% CI: -1.3 to -0.3, p = 0.001) were associated with poorer oral care attitude. Regarding work settings, better attitude was reported in those assigned to ICU (β = 1.7, 95% CI: 1.1 to 2.2, p<0.001) and other departments, such as psychiatric units (β = 0.7, 95% CI: 0.1 to 1.3 p = 0.022), compared to those in medical, stroke and geriatric departments. Regarding oral care guideline training, a lower level of attitude towards oral care was shown in the health care professionals who had not learned oral care guidelines in training (β = -0.6, 95% CI: -0.9 to -0.2, p = 0.001) compared with those who did. Better oral care knowledge was associated with better oral care attitude (β = 0.1, 95% CI: 0.0 to 0.1, p = 0.005).

In the model of oral care practice (R^2^ = 0.3), a significant relationship was shown among practice and work positions (p<0.001), oral care guideline training (p<0.001), oral care knowledge (p<0.001) and oral care attitude (p<0.001). Regarding work positions, EN had better oral care practice compared with RN (β = 0.9, 95% CI: 0.4 to 1.3, p<0.001). Regarding oral care guideline training, a lower level of oral care practice was shown in the nursing staff who had not learned oral care guidelines in training (β = -0.6, 95% CI: -1.0 to -0.3, p<0.001) compared with those who did. Better oral care practice was associated with higher levels of oral care knowledge (β = 0.1, 95% CI: 0.1 to 0.1, p<0.001) and oral care attitude (β = 0.3, 95% CI: 0.2 to 0.4, p<0.001).

## Discussion

This cross-sectional survey provided a comprehensive assessment of oral care KAP, training needs and experiences among nursing staff in various wards of acute hospital settings in Hong Kong. The level of oral care KAP of the nursing staff appeared to be relatively low. The RN had the highest oral care knowledge, where EN had the highest oral care attitude and practice. On the other hand, HCA had the lowest oral care knowledge and practice scores. Most of the nursing staff perceived the need to receive updates on oral care and had unpleasant oral care experience.

### Oral care practice

As with the literature [12,13,26,28,29], our results showed that nursing staff did not carry out evidence-based oral care practice. We found that oral care guideline training and knowledge were associated with higher level of oral care practice, inspiring future studies to improve practice through training and knowledge transfer. Moreover, the standard of oral care practice can be further improved and enhanced by removing such barriers for example, resources allocation, communication with patients and supervisors.

Our findings also reveal that few nursing staff in our study conducted oral health assessments upon admission. When oral care assessment was not performed, oral care practices appeared to be inadequate [27, 41]. Furthermore, respondents in our study reflected that oral care guidelines or protocols were not easily available at work, which could explain the mean score of 5.4 out of 10 for the usefulness of oral care guideline to improve oral care practice. These results suggest the importance of the availability and accessibility of oral care protocols in work settings.

### Oral care knowledge

The lack of oral care knowledge among the nursing staff as revealed by our results is consistent with previous research findings [12, 25, 40, 44]. While some studies described oral care knowledge in terms of summary scores [25, 26, 34, 40], only two further categorised the scores [34, 40]. Moreover, the studies that complied with composite scores only focused on the ICU settings or the oncology wards [25, 26, 34, 40]. The composite scores used in our study comprehensively covered aspects not restricted to particular wards or settings, and the consistent cut-off values would facilitate cross study comparisons in future.

We found oral care guideline training and unpleasant experience associated with higher oral care knowledge level. Literature also reported oral care guidelines could help to standardise oral care knowledge and training [14, 29, 34, 36]. Although training was associated with higher knowledge level, over half of our respondents did not learn oral care guidelines during their training. Future trainings would consider incorporating oral care guideline in the formal training.

### Oral care attitude

We found oral care guideline training, unpleasant experience and oral care knowledge were associated with higher level of attitude towards providing oral care to patients. In the current study, the nursing staff who did not undergo oral care guideline training had lower level of oral care attitude. Various studies revealed that with adequate training, nursing staff could show high priority in oral care [17, 39]. Hence, future trainings should emphasize the importance of oral care and include oral care components in the priority list of nursing care.

### Training preference on oral care

While the majority of our respondents learned oral care knowledge in their nursing school training, they might not have enough in-depth knowledge on the possible adverse effects of poor oral health, drugs with adverse effects on oral health and the benefits of oral care practice. The training curriculum without adequate emphasis on oral care could be one of the reasons explaining their oral care knowledge deficit. Studies in other places also found oral care knowledge was not the main focus in the curriculum [14, 25, 40]. In addition, relevant knowledge has not been reinforced and updated in the existing continuing training and education programmes [26]. Therefore, nursing staff might not be able to receive updated clinical oral care knowledge.

Most of our study respondents perceived a need to receive annual updates on oral care through on-the-job training, with oral care procedure as the most important area. Future training on oral care procedures and the use of oral care assessment scale may be developed in accordance with the preference of the nursing staff to motivate participation. Oral care is important not only to patients in the ICU or oncology ward settings, but also patients in other settings. Revamping training curricula, promoting knowledge update, evidence-based practice, oral care assessment and protocols are possible solutions to address the problem of oral care knowledge deficit and inadequate oral care practice [13]. Furthermore, oral care protocols for patients should be constructed for the establishment of standardised oral care policies which can promote formalisation and uniform oral care provision. This can address the gap between actual practices and variations of policies among different organisations [13].

### Unpleasant oral care experience

Our study adds to the literature that nursing staff with unpleasant experiences have higher level of oral care knowledge and attitude. It might be speculated that after the unpleasant experiences, the nursing staff proactively acquired relevant knowledge in order to avoid similar unpleasant experience again. Coaching or debriefing with staff having unpleasant experience might be offered to support their pursuit of appropriate oral care knowledge. Oral care knowledge and experience were significantly associated, so did between oral care practice and knowledge, which in turn could result in indirect effect that experience resulted in better knowledge and better knowledge resulted in better practice. However, with a cross-sectional design, we could not verify this speculation. Further studies, qualitative studies in particular, to explore such relationship are warranted.

The lack of time and manpower was reflected by the respondents to be the most common barriers for their performance of oral care practice, which are consistent with the results of previous studies [11, 25, 27, 34, 35]. Furthermore, patients’ behaviour was another common barrier to oral care performance. Similarly, uncooperative patients were regarded as the main barrier to oral care practice [12, 27, 28]. To remove such barrier, communication between patients, family caregivers and the nursing staff should be strengthened to enhance cooperation from the patients and their caregivers.

### Strengths and limitations

The strength of this study is the development of a questionnaire by consolidating evidence from different studies and clinical expertise. While previous studies also employed composite scores [15, 26, 36], our tool has a more comprehensive coverage, clearer scoring system and cut-off values for classification, such that our tool could enhance comparison across studies. Nevertheless, there are some limitations. The variable gender was not collected in this survey because gender was rarely considered as a factor to study oral care KAP [27, 37, 45]. The study could benefit from including gender as an explanatory variable for oral care KAP. While convenience sampling was widely used in similar studies [24, 25, 34], the use of a non-random sample might limit the external validity of this study results and its generalization. Probability sampling could be adopted in future studies. The cross-sectional nature of the study could not investigate causation.

### Clinical implication and recommendation

Future studies on oral care KAP should consider the use of a standardized and comprehensive instrument, with consistent cut-offs for classifications, such as the one developed in this study. Availability and accessibility of oral care protocols, training on updated clinical oral care knowledge, oral care practice procedure, use of protocol and oral care assessment scale, communication with patients, family and nursing staff, and interventional studies are suggested directions for future research and clinical developments. Training components should be enriched with such communication skills.

## Conclusion

The nursing staff in acute hospital settings generally have poor KAP towards oral care, with the RN having the highest oral care knowledge, EN had the highest oral care attitude and practice, while HCA had the lowest oral care knowledge and practice. Only 14.9% of the nursing staff were rated as having good oral care practice (RN: 16.4%; EN: 20.2%; HCA: 7.0%), whereas 53.2% of them (RN: 41.8%; EN: 67.9%; HCA: 66%) had unpleasant oral care experience. This study adds to the literature the association between oral care unpleasant experiences and the oral care practice, as well as oral care knowledge and attitude which also in turns associated with practice. These findings shed light on the oral care training and guidelines development in future. The developed standardised tool could also be applied for future studies. Recommendations on the future research, training and practices were made.

## Supporting information

S1 Appendix Summary of the revised items in the draft of the questionnaire from the focus group findings. (PDF)

S2 Appendix Oral care knowledge, attitude and practice question. (PDF)

S1 Data (XLSX)
