# Supplementary material for: Oral care knowledge, attitude and practice among nursing staff in acute hospital settings in Hong Kong
Source: PLoS One. 2023 Aug 15;18(8):e0289953. doi: 10.1371/journal.pone.0289953 (PMC10427009; doi:10.1371/journal.pone.0289953)
Supplement: S1 Appendix — (PDF) [file pone.0289953.s001.pdf]

Appendix 1. Summary of the revised items in the draft of the questionnaire from the focus group findings

No	Addition / Deletion	Revised details	Reasons for the revised items
1.2	Addition	Question about: "how much time is needed for each oral cavity cleansing?" is added.	Time needed for each oral care was different from different nursing staff in focus groups. Therefore, this question can be included for assessing nursing staff's oral care practice.
1.11	Addition	Question about "usefulness of oral care guideline or protocol for improving the practice of oral care" is added.	Lack of oral care assessment or protocol for improving the oral care practice was reported from the findings of focus group. Therefore, this question can be included for assessing nursing staff's oral care knowledge and practice.
2.1	Addition	Question about "the kinds of patients who need oral care cleansing" is added.	Lack of knowledge was reported from the focus group findings. This question can be included for assessing nursing staff's oral care knowledge.
2.4	Deletion	Question about toothette is deleted.	Not all nursing staff in focus group agreed the usefulness of toothette and not all working departments had this tool for oral cleansing. Therefore, this question can be deleted.
2.8	Addition	Question about "the possible benefits of oral care practice" is added.	Lack of knowledge was reported from the focus group findings. This question can be included for assessing nursing staff's oral care knowledge.
4.4	Addition	Question about "the kind of oral care knowledge nursing staff need to update" is added.	Lack of resources on oral care education, training and promotion was reported from the focus group findings. This question can be included for assessing nursing staff's oral care training.
5.1 to 5.6	Addition	Questions about "unpleasant experience of performing oral care" are added.	Difficulties to oral care were reported from the focus group findings. This question can be included for assessing nursing staff's barriers to oral care.
