# Supplementary material for: Oral care knowledge, attitude and practice among nursing staff in acute hospital settings in Hong Kong
Source: PLoS One. 2023 Aug 15;18(8):e0289953. doi: 10.1371/journal.pone.0289953 (PMC10427009; doi:10.1371/journal.pone.0289953)
Supplement: S2 Appendix — (PDF) [file pone.0289953.s002.pdf]

Appendix 2. Oral care knowledge, attitude and practice questionnaire

Screening
篩選

S1. Are you a nursing staff (registered nurse, enrolled nurse, nursing assistant, healthcare assistant)?

你是否護理人員（註冊護士，登記護士，護士助理員，健康服務助理員）？

- □ Yes → Please proceed to S2.
- □ 是 → 請回答 S2.
- □ No → Thank you for your participation, this is the end of the questionnaire.
- □ 否 →感謝您的參與，此問卷調查完畢。

S2. Are you currently working at a hospital setting in Hong Kong?
你目前是否在香港的醫院裡工作？

- □ Yes → Please proceed to S3.
- □ 是 → 請回答 S3.
- □ No → Thank you for your participation, this is the end of the questionnaire.
- □ 否 →感謝您的參與，此問卷調查完畢。

S3. Are you currently working at a department or ward in acute setting? In this regard, outpatient departments, palliative wards and rehabilitation wards are not considered as acute setting.
你目前工作的地方會否處理需要急性住院或護理的病人？在這點上，門診、舒緩治療和康復並不屬於急性護理。

- □ Yes → Please proceed to S4.
- □ 是 → 請回答 S4.
- □ No → Thank you for your participation, this is the end of the questionnaire.
- □ 否 →感謝您的參與，此問卷調查完畢。

S4. Do you need to provide routine oral care to patients in your health care setting? Please note that their need of oral care is assessed, determined and initiated by nursing staff, not upon request by patients.
在你工作的病房裡，是否需要為病人提供恆常口腔護理？請留意，他們的口腔護理需要是由護理人員進行評估、確定和實行，而不是由病人所提出。

- □ Yes → Please proceed to Q1.1.
- □ 有 → 請回答 Q1.1.
- □ No → Thank you for your participation, this is the end of the questionnaire.
- □ 否 →感謝您的參與，此問卷調查完畢。

NOTE: For the rest of this questionnaire, "patients" refers to those who need oral care, their need of oral care is assessed, determined and initiated by nursing staff, not upon request by patients.
注意：本問卷的餘下部分，"病人"是指需要接受口腔護理的患者，而他們的口腔護理需要是由護理人員進行評估、確定和實行，而不是由病人所提出。
Part I Practice of oral care for patients at hospital
第一部分　口腔護理的實踐

1.1 How often is oral cavity cleansing performed for patients at your health care setting? (please select the MOST common frequency)
在你工作的環境裡，多久為病人進行口腔清潔？（請選擇最普遍的情況）

- □ More than twice a day 每日多於兩次
- □ Twice a day 每日兩次
- □ Once a day 每日一次
- □ Once every two days 每兩日一次
- □ Once a week 每星期一次
- □ When there is oral/dental problem 每當有口腔/牙齒問題
- □ Never 從來沒有
- □ Varies (depending on the condition of the patient) 不定期（取決於病人的狀況）
- □ Other, please specify: 其他，請列明：

1.2 How many patients do you need to provide oral care per day?
你每天需要為多少患者提供口腔護理？

- □ Less than five 少於五位
- □ Five to ten 五至十位
- □ Eleven to fifteen 十一至十五位
- □ Sixteen to twenty 十六至二十位
- □ More than twenty 多於二十位
- □ Other, please specify: 其他，請列明：

1.3 How much time is needed for each oral cavity cleansing?
每次口腔清潔需要多少時間？

- □ Less than one minute 少於一分鐘
- □ Around two to five minutes 大約兩到五分鐘
- □ Around six to ten minutes 大約六到十分鐘
- □ More than ten minutes 超過十分鐘
- □ Varies (depending on the condition of the patient) 不定時（取決於病人的狀況）
- □ Other, please specify: 其他，請列明：

1.4 What is (are) the tool(s) used for the oral cavity cleansing for patients at your health care setting? (multiple answers allowed)
在你工作的環境裡，會用什麼用具為病人進行口腔清潔？（可多於一個答案）

- □ Oral cavity cleansing not performed 不會進行口腔清潔
- □ Manual toothbrush 手動牙刷
- □ Electric toothbrush 電動牙刷
- □ Forceps and gauze 鉗和紗布
- □ Spatulas and gauze 大平鉗和紗布
- □ Large cotton stick 大棉花棒
- □ Toothette 海棉棒
- □ Floss stick 牙線棒

- □ Suctioning equipment 吸痰設備
- □ Other, please specify: 其他，請列明：

1.5 What is (are) the agent(s) used for the oral cavity cleansing for patients at your health care setting? (multiple answers allowed)
在你工作的環境裡，會用什麼口腔清潔劑為病人進行口腔清潔？（可多於一個答案）

- □ Oral cavity cleansing not performed 不會進行口腔清潔
- □ Water 清水
- □ Toothpaste 牙膏
- □ 0.9% sodium chloride 0.9%氯化鈉
- □ Glycothymoline or diluted thymol gargle 稀釋的漱口水
- □ None 沒有
- □ Other, please specify: 其他，請列明：

1.6 Other than oral cavity cleansing, what type(s) of oral care is (are) provided to the patients in your health care setting? (multiple answers allowed)
在你工作的環境裡，除了口腔清潔，會替病人進行其他口腔護理嗎？（可多於一個答案）

- □ Denture cleaning 假牙清潔
- □ Oral health assessment 口腔健康評估
- □ Report to doctors for referral to dentists 報告予醫生從而轉介給牙醫
- □ Other, please specify: 其他，請列明：
- □ No any other oral care 沒有其他口腔護理

1.7 Do patients receive an oral health assessment in your health care setting? Please note that definition of oral health assessment is assessing cleanliness and abnormalities of oral cavity, e.g. tooth decay.
在你工作的環境裡，病人有否接受口腔健康評估？請留意，口腔健康評估是評估口腔是否清潔和異常，例如：蛀牙。

- □ Yes, always 有，經常
- □ Yes, sometimes 有，有時候
- □ Yes, rarely 有，很少
- □ No, never 沒有，從來沒有

1.8 When is oral health assessment provided (if any) in your health care setting?
在你工作的環境裡，何時會提供口腔健康評估(如適用)？

- □ Oral health assessment not provided 不會提供口腔健康評估
- □ On admission 入院時
- □ During hospitalization 住院期間
- □ Before discharge 出院前
- □ No fixed pattern 沒有特定的時間
- □ Other, please specify: 其他，請列明：

1.9 Who carries out most of the oral care for patients in your health care setting? (multiple answers allowed)
在你工作的環境裡，誰會替病人執行大部分的口腔護理？（可多於一個答案）

- □ Registered nurses 註冊護士
- □ Enrolled nurses 登記護士
- □ Nursing assistants 護士助理員
- □ Healthcare assistants 健康服務助理員
- □ Family caregivers 家人照顧者
- □ Other, please specify: 其他，請列明：

1.10 Are you directly involved in (e.g. hands-on) oral care delivery to patients in your health care setting? Please note giving instructions to other staff is NOT considered as "directly involved"
在你工作的環境裡，是否直接參與(例如：親手)為病人提供的口腔護理？請留意，向其他工作人員發出指示並不代表「直接參與」。

- □ Yes, always 有，經常
- □ Yes, sometimes 有，有時候
- □ No 沒有

1.11 Are there any guidelines for oral care procedures for patients in your health care setting?
在你工作的環境裡有沒有口腔護理程序的指引？

- □ Yes, they are: 有，它們是
- □ No 沒有
- □ Not sure 不肯定

1.12 Do you use oral care guideline or protocol in your health care setting?
在你工作的環境裡，你有沒有使用口腔護理指引？

- □ Yes 有
- □ No 沒有

1.13 Do you think oral care guideline or protocol is useful for improving the practice of oral care?
Please rate the level of usefulness on a scale of 0 to 10 (Higher score indicates higher level of usefulness) ____
你認為口腔護理指引能有效改善口腔護理的實踐嗎？請以 0 到 10 評價口腔護理指引的效用（分數越高表示效用越高）____

1.14 Are there any problems which hinder you from performing oral care at your health care setting?
在你工作的環境裡，有什麼問題阻礙你執行口腔護理？

- □ Yes, please specify (multiple answers allowed) 有，請列明：（可多於一個答案）
  - □ Not enough time 沒有足夠時間
  - □ Not enough manpower 沒有足夠人手
  - □ Not enough training 沒有足夠陪訓
  - □ Not enough tools/equipment 沒有足夠用具/設備

- □ Not enough confidence to perform 沒有足夠信心執行
- □ Not enough skill to perform 沒有足夠技巧執行
- □ No protocol/guideline 沒有條約/指引
- □ Patients' physical condition (e.g. on ventilation) 病人的身體狀況(例如利用呼吸機呼吸）
- □ Patients' behavior (e.g. refuse opening mouth) 病人的行為（例如拒絕張開口）
- □ Patients' attitude (e.g. refuse oral care) 病人的態度（例如拒絕口腔護理）
- □ Perceived as a disgusting duty 被視為一種令人厭惡的工作
- □ Afraid of being bitten by the patient 擔心被病人咬傷
- □ Afraid of deviating from current protocol/practice 擔心偏離現時的條約/習慣
- □ Afraid of dislodging the medical equipment on the patient 擔心遺留醫療用具在病人身上
- □ Afraid of side effects/complications (e.g. bleeding, tooth accidentally swallowed or aspirated by patient) 擔心副作用/併發症(例如出血，牙齒不小心被病人吞下或吸入）
- □ Afraid of being complained by relatives of patients 擔心被病人親屬投訴
- □ Afraid of additional charges on patients 擔心要負擔額外醫療開支
- □ Unreasonable requests by relatives of patients 病人親屬無理的要求
- □ It is not important 口腔護理並不重要
- □ It is not my duty 口腔護理並不是我工作的範疇
- □ Other, please specify 其他，請列明：

□ No 沒有

Part II Knowledge of oral care for patients at hospital
第二部分　口腔護理的知識

2.1 What kind(s) of patients need(s) oral care cleansing? (multiple answers allowed)
什麼病人需要口腔護理清潔？（可多於一個答案）

- □ Patient with pneumonia 肺炎患者
- □ Patient with tracheostomy 有氣管造口的患者
- □ Patient on Ryle's tube feeding 有鼻胃管的患者
- □ Patient on ventilator 呼吸機患者
- □ Not sure 不肯定
- □ Other, please specify: 其他，請列明：

2.2 In oral health assessment, what is/are indicator(s) of poor oral health? (multiple answers allowed)
在口腔健康評估中，你認為哪些是口腔健康不良的指標？（可多於一個答案）

- □ Tooth appearance 牙齒外觀
- □ Bad breath 口臭

- □ Gum bleeding 牙齦出血
- □ Ulcer 潰瘍
- □ Loose teeth 牙齒鬆動
- □ Tooth decay (cavities) 蛀牙
- □ Dental plaque deposition 牙垢沉積
- □ Sputum retention 痰液滯留
- □ Abscess 膿腫
- □ Previous / current oral diseases 之前/現有的口腔疾病
- □ Not sure 不肯定
- □ Other, please specify: 其他，請列明：

2.3 What is/are the possible adverse effect(s) of poor oral health? (multiple answers allowed)
不良的口腔健康會帶來什麼負面影響？（可多於一個答案）

- □ Gum diseases 牙齦疾病
- □ Hypertension 高血壓
- □ Cardiovascular diseases 心血管病
- □ Stroke 中風
- □ Fracture 骨折
- □ Diabetic complications 糖尿病併發症
- □ Fungal oral infections 真菌口腔感染
- □ Pneumonia 肺炎
- □ Malnutrition 營養不良
- □ Not sure 不肯定
- □ Other, please specify: 其他，請列明：

2.4 Are you aware of any drugs which adversely affect oral health?
你是否知道哪些藥物會對口腔健康造成不利影響？

- □ Yes, they are: 有，它們是
- □ No 沒有
- □ Not sure 不肯定

2.5 Have you ever used or heard of any oral care assessment scale?
你有否用過或聽過口腔護理評估量表？

- □ Yes, used, please name the scale: 有，用過，請列明：
- □ Yes, heard, please name the scale: 有，聽過，請列明：
- □ No 沒有

2.6 How confident are you in performing oral care procedures for patients? Please rate the level of confident on a scale of 0 to 10 (Higher score indicates higher level of confident) ____
你有多少信心為病人進行口腔護理的程序？請以 0 到 10 評價為病人進行口腔護理程序的信心（分數越高表示信心越高）____

2.7 How confident are you in answering questions about oral care from patients and/or their family caregivers? Please rate the level of confident on a scale of 0 to 10 (Higher score indicates higher level of confident) ____
你有多少信心回答病人/及其家人關於口腔護理的問題？請以 0 到 10 評價為回答病人/及其家人關於口腔護理問題的信心（分數越高表示信心越高）____

2.8 What is/are the possible benefit(s) of oral care practice? (multiple answers allowed)
口腔護理為病人帶來什麼好處？（可多於一個答案）
- □ Improved oral health 改善口腔健康
- □ Improved personal image 改善個人形象
- □ Improved appetite 改善食慾
- □ Improved drug compliance 改善藥物依從性
- □ Decreased health complications from poor oral health 減少口腔健康不良所帶來的併發症
- □ Not sure 不肯定
- □ Other, please specify: 其他，請列明：

Part III Perceived needs of oral care of patients in hospitals
第三部分 口腔護理的認知需要

3.1 What is (are) the reason(s) of performing oral care? (multiple answers allowed)
你進行口腔護理的原因是什麼？（可多於一個答案）
- □ Maintaining hygiene 保持口腔衛生
- □ Promoting comfort 促進舒適
- □ Maintaining patients' dignity 保持病人的尊嚴
- □ Preventing infection 預防感染
- □ Following protocol/guideline 跟隨條約/指引
- □ Patients'/Carers' request 病人/其家人的要求
- □ Other, please specify: 其他，請列明：

3.2 With regard to the daily duties required of nursing staff, please rate the level of priority given to oral care on a scale of 0 to 10 (Higher score indicates higher priority) ____
關於護理人員所需的日常工作，請以 0 到 10 評價口腔護理的優先次序（分數越高表示次序越高）____

3.3 How often do you think that oral cavity cleansing should be performed? (please select the MOST common frequency)
你認為多久應為病人進行口腔清潔？（請選擇<u>最</u>普遍的情況）
- □ Twice a day 每日兩次
- □ Once a day 每日一次
- □ Every two days 每兩日一次
- □ Once a week 每星期一次
- □ When there is problem 每當有口腔/牙齒問題
- □ Varies (depending on the condition of the patient) 不定期（取決於病人的狀況）

- □ No need 不需要
- □ Other, please specify: 其他，請列明：

3.4 Do you think all patients should have oral assessment on admission?
你會否認為所有病人應該在入院時需要進行口腔評估？

- □ Strongly necessary 十分需要
- □ Necessary 需要
- □ Not sure 不肯定
- □ Not necessary 不需要
- □ Strongly not necessary 十分不需要

3.5 Who do you consider to be the major responsible staff for patients' oral care?
你認為病人的口腔護理應主要由誰負責？

- □ Registered nurse 註冊護士
- □ Enrolled nurse 登記護士
- □ Nursing assistant 護士助理員
- □ Healthcare assistant 健康服務助理員
- □ Family caregiver 家人照顧者
- □ Volunteer 義工
- □ Other, please specify: 其他，請列明：

Part IV Training in oral care delivery
第四部分 口腔護理培訓

4.1 Where did you learn oral care delivery? (multiple answers allowed)
你曾經在什麼機構學習口腔護理（可多於一個答案）

- □ At nursing school (Baccalaureate degree) 護士學校（學士學位）
- □ At nursing school (Higher diploma/ diploma) 護士學校（高級文憑/文憑）
- □ Pre-job training 工作前培訓
- □ On-the-job training 工作培訓
- □ CNE courses 護士持續進修課程
- □ Self-directed learning 自修學習
- □ Other, please specify:

4.2 Have you learned any guidelines about oral care in your training?
在訓練中，你有沒有學習有關口腔護理的指引？

- □ Yes, please specify: 有，請列明：
- □ No 沒有
- □ Other, please specify: 其他，請列明：

4.3 Do you think nursing staff need to receive updates on oral care?
你會否認為護理人員需要接收口腔護理的更新知識？

- □ Yes, how often is the update 需要，多久需要更新
    - □ Every quarter 每個季度
    - □ Every year 每年

- □ Every rotation of ward 每次病房轉換
- □ Other, please specify: 其他，請列明：

□ No 不需要

4.4 Which kind of oral care knowledge do you think nursing staff need to update? (multiple answers allowed)
你認為護理人員需要更新哪一種口腔護理知識？（可多於一個答案）

- □ Oral assessment guide 口腔評估指引
- □ Oral care assessment scale 口腔護理評估量表
- □ Oral care procedure 口腔護理的程序
- □ Oral documentation 口腔護理的記錄
- □ Oral care theory 口腔護理的理論
- □ Other, please specify: 其他，請列明：

4.5 If post-qualification training on oral care is to be provided, which format of the training would you prefer?
如果提供專業訓練後的口腔護理培訓，你會選擇哪一種的培訓方式？

- □ Pre-job training 工作前培訓
- □ On-the-job training 工作培訓
- □ Attend courses 參加課程
- □ E-learning 網上課程
- □ Other, please specify: 其他，請列明：

4.6 Do you think it would be useful to have an oral assessment guide?
你是否認為口腔評估指引是有用嗎？

- □ Very useful 十分有用
- □ Useful 有用
- □ Not sure 不肯定
- □ Not useful 沒有用
- □ Strongly not useful 十分沒有用

Part V Oral care experience of the nursing staff
第五部分 護理人員的口腔護理經驗

5.1 Do you have any unpleasant experience in performing oral care for patients?
你為病人進行口腔護理時，曾經有什麼不愉快的經歷嗎？

- □ Yes, please specify (multiple answers allowed) 有，請列明：（可多於一個答案）
    - □ Refused by patients 受到病人拒絕
    - □ Scolded by patients 受到病人責罵
    - □ Poor behavior from patients 病人態度惡劣
    - □ Non-compliance from patients 病人不服從
    - □ Hurt by patients 被病人弄傷
    - □ Injured patients 使病人受傷
    - □ Not supported by supervisors or colleagues 不受上司或同事的支持

- □ Complained by patients 受到病人投訴
- □ Complained by relatives of patients 受到病人家屬投訴
- □ Other, please specify: 其他，請列明：

□ No 沒有

5.2 Do you know the reason(s) of refusing oral care by patients?
你是否知道病人拒絕口腔護理的原因嗎？

□ Yes, please specify (multiple answers allowed) 是，請列明：（可多於一個答案）

- □ Oral care is not important 口腔護理並不重要
- □ Worrying about financial problem 擔心金錢問題
- □ Worrying about issue of dignity 擔心尊嚴的問題
- □ Fear of pain 害怕痛苦
- □ Fear of choking 害怕窒息
- □ Taste of cleansing agent 口腔清潔劑的味道
- □ Other, please specify: 其他，請列明：

□ No 否

□ Not applicable (Not experienced) 不適用（沒有相關經歷）

5.3 Do you know the reason(s) of oral care not being supported by supervisors or colleagues?
你是否知道口腔護理不受上司或同事的支持的原因嗎？

□ Yes, please specify (multiple answers allowed) 是，請列明：（可多於一個答案）

- □ Low priority of oral care 口腔護理不是優先處理
- □ Oral care is not important 口腔護理並不重要
- □ Oral care is not your own responsibility 口腔護理並不是你的責任
- □ Lack of resources 缺乏資源
- □ Lack of time 缺乏時間
- □ Lack of oral care training 缺乏口腔護理訓練
- □ Complained by patients or relatives of patients 受到病人或病人家屬投訴
- □ Other, please specify: 其他，請列明：

□ No 否

□ Not applicable (Not experienced) 不適用（沒有相關經歷）

5.4 Do you know the reason(s) of complaints by relatives of patients?
你是否知道家屬投訴的原因嗎？

□ Yes, please specify (multiple answers allowed) 是，請列明：（可多於一個答案）

- □ Not satisfy the oral care performance by nursing staff 家屬不滿意護理人員所執行的口腔護理表現
- □ Worrying about financial problem 擔心金錢問題

- □ Worrying about issue of dignity 擔心尊嚴的問題
- □ Worrying the patients may suffer from pain during the cleansing 擔心在口腔護理過程中，病人遭受痛苦
- □ Worrying the patients may suffer from choking during the cleansing 擔心在口腔護理過程中，病人遭受窒息
- □ Other, please specify: 其他，請列明：

- □ No 否
- □ Not applicable (Not experienced) 不適用（沒有相關經歷）

5.5 Do you think the unpleasant experience will hinder your oral care practice?
你是否認為這些不愉快的經歷會阻礙你為病人進行口腔護理嗎？

- □ Totally hinder 完全阻礙
- □ Hinder 阻礙
- □ Not sure 不肯定
- □ Not hinder 沒有阻礙
- □ Totally not hinder 完全沒有阻礙
- □ Not applicable (Not experienced) 不適用（沒有相關經歷）

5.6 Do you think the unpleasant experience will affect your attitude on oral care practice?
你是否認為這些不愉快的經歷會影響你對口腔護理的態度嗎？

- □ Totally affect 完全影響
- □ Affect 影響
- □ Not sure 不肯定
- □ Not affect 沒有影響
- □ Totally not affect 完全沒有影響

Part V Profile of the nursing staff
第六部分 護理人員的背景概述

6.1 What is your present position?
你現在的職級是什麼？

- □ General Manager (GM)/ Department Operations Manager (DOM) 總經理/部門運作經理
- □ Nurse consultant 顧問護士
- □ Ward manager 病房經理
- □ Nurse specialist 專科護士
- □ Advanced practice nurse 資深護師
- □ Registered nurse 註冊護士
- □ Enrolled nurse 登記護士
- □ Nursing assistant 護士助理員
- □ Healthcare assistant 健康服務助理員
- □ Other, please specify: 其他，請列明：

6.2 What is your <u>first</u> qualification training?
你<u>第一個</u>培訓資歷是什麼？

- □ Registered nurse (Baccalaureate degree) 註冊護士（學士學位）
- □ Registered nurse (Higher diploma/ diploma) 註冊護士（高級文憑/文憑）
- □ Enrolled nurse 登記護士
- □ Nursing assistant 護士助理員
- □ Healthcare assistant 健康服務助理員
- □ Other, please specify: 其他，請列明：

6.3 How long have you been qualified (the <u>first</u> qualification)? _________ years
你已擁有培訓資歷多少年？（<u>第一個</u>培訓資歷）____________年

6.4 Where did you receive your qualification training (the <u>first</u> qualification)?
你在哪裡獲得培訓資歷（<u>第一個</u>培訓資歷）？
- □ Hong Kong 香港
- □ Mainland China 中國
- □ Other, please specify: 其他，請列明：

6.5 What is your <u>latest</u> qualification training?
你<u>最新</u>的培訓資歷是什麼？
- □ Registered nurse (Baccalaureate degree) 註冊護士（學士學位）
- □ Registered nurse (Higher diploma/ diploma) 註冊護士（高級文憑/文憑）
- □ Enrolled nurse 登記護士
- □ Nursing assistant 護士助理員
- □ Healthcare assistant 健康服務助理員
- □ Other, please specify: 其他，請列明：

6.6 How long have you been qualified (the <u>latest</u> qualification)? _____ years
你已擁有培訓資歷多少年？（<u>最新</u>培訓資歷）____________年

6.7 Where did you receive your qualification training (the <u>latest</u> qualification)?
你在哪裡獲得培訓資歷（<u>最新</u>培訓資歷）？
- □ Hong Kong 香港
- □ Mainland China 中國
- □ Other, please specify: 其他，請列明：

6.8 What is your highest education attainment?
你最高的學歷是什麼？
- □ Doctoral or above 博士或以上
- □ Master 碩士
- □ Degree 學士
- □ Associate Degree 副學士
- □ Higher Diploma 高級文憑
- □ Diploma 文憑
- □ Certificate 證書
- □ Other, please specify: 其他，請列明：

6.9 What is your current work setting?
你現在的工作環境是什麼？
- □ Acute medical ward 急症內科病房
- □ Acute medical and geriatric ward 急症內科及老人科病房
- □ Acute stroke ward 急症中風病房

- □ Orthopedics ward 骨科病房
- □ Acute surgical ward 急症外科病房
- □ General surgical ward 普通外科病房
- □ Psychiatric ward 精神科病房
- □ Intensive Care Unit 深切治療病房
- □ Other, please specify: 其他，請列明：

6.10 What is your shift arrangement?
你工作的時間是什麼？

- □ Internal rotation 內部輪班
- □ Long term night duty 長期夜班
- □ Day duty 日班
- □ Office hours 辦公時間
- □ Other, please specify 其他，請列明：

6.11 How many years of working experience in the current setting/ current position? _____Years
你在目前工作的地方/當前職位有多少年工作經驗？_____年

6.12 Is your current work setting publicly-funded or privately funded?
你目前的工作環境是政府資助或是私人資助？

- □ Public 政府資助
- □ Private 私人資助
- □ Other, please specify: 其他，請列明：

<< End of questionnaire, thank you very much >>
<<問卷調查結束，多謝您的參與>>
